# Identification of *CD320*-Associated Cobalamin Transport Deficiency Through Newborn Screening Caused by Novel Homozygous Candidate Variants

**DOI:** 10.3390/genes17091108

**Published:** 2026-09-12

**Authors:** Faisal Almalki, Amal Saleh Alsofyany, Abdulaziz K. Almalki, Ashraf Oudah Abu Shuaylan, Haya Alruqi, Jwaher Mesleh Althobiti

**Affiliations:** 1 Clinical Laboratory Sciences, Applied Medical Science, Taibah University, Medina 42353, Saudi Arabia; 2 Center for Genetics and Inherited Diseases, Taibah University, Medina 42318, Saudi Arabia; hsruqi@taibahu.edu.sa; 3 Alhada Armed Forces Hospital, Ministry of Defence, Taif 26792, Saudi Arabia; a_s_aksa@hotmail.com (A.S.A.); almalkiabdulaziz@hotmail.com (A.K.A.); ash.oudah@gmail.com (A.O.A.S.)

**Keywords:** transcobalamin receptor impairment, methylmalonic acid (MMA), propionylcarnitine (C3), *CD320*, novel, missense variant, consanguineous

## Abstract

Background: The transcobalamin receptor, encoded by the *CD320* gene, mediates the cellular uptake of the transcobalamin–cobalamin complex (vitamin B_12_). Pathogenic variants in *CD320* impair the intracellular transport, resulting in methylmalonic acid (MMA) and propionylcarnitine (C3) level, despite normal circulating vitamin B_12_ levels, with variable clinical phenotypes. Methods: Whole-exome sequencing (WES) was carried out for a newborn screening positive proband. Further plasma metabolic investigation and blood gas analysis were carried out for the proband. Sanger sequencing was carried out to confirm the candidate variant and identify the familial segregation. Results: The proband presented with elevated C3 and MMA levels and exhibited an expanded clinical phenotype that included severe microcephaly, metabolic acidosis, low bicarbonate and pancytopenia. WES identified a homozygous missense candidate variant in the last amino acid of exon 1 in the *CD320* gene (NM_016579.3: c.142G>C, p.Gly48Arg). Another adjacent synonymous homozygous candidate variant was identified (NM_016579.3: c.141A>C, p.Ala47=). Both variants were identified in a male proband from a consanguineous family. Both variants were confirmed by Sanger and segregated within the family members. During one year of follow-up, the patient showed persistent microcephaly while receiving a specialized metabolic formula and levocarnitine supplementation. Conclusions: This study identified a novel recessive missense candidate variant and an adjacent synonymous candidate variant in *CD320*, supported with biochemical and clinical features of *CD320*-associated cobalamin transport deficiency. This suggests microcephaly may be a previously unrecognized phenotype with possible association of impaired intracellular cobalamin transport, while elevated C3 detected through routine newborn screening may be the earliest biochemical indicator of *CD320*-associated cobalamin transport deficiency.

## 1. Introduction

Cobalamin (or vitamin B_12_) is an important water-soluble vitamin that is required for neurological functions, DNA synthesis, and erythropoiesis [1]. After cobalamin is absorbed in the intestine, it binds to intrinsic factor and is taken up by enterocytes. It then associates with transcobalamin in the bloodstream to form a transcobalamin–cobalamin complex, facilitating its transport to peripheral tissues. Cellular uptake of this complex is carried out by the transcobalamin receptor, which is encoded by the *CD320* gene, through receptor-mediated endocytosis [2].

The *CD320* gene (OMIM: 606475) is located at 19p13.2 and comprises five exons. A pathogenic variant in the *CD320* gene was found to impaired cellular cobalamin uptake [3]. Impaired receptor function, due to the disturbance of the cobalamin-dependent metabolic pathway, reduces cellular cobalamin availability despite normal circulating vitamin B_12_ levels [3,4]. The initial *CD320* variant was identified in newborns with elevated methylmalonic acid (MMA) and propionylcarnitine (C3) detected via newborn screening (NBS) [3]. Functional studies of patient-derived fibroblasts showed reduced binding and uptake of the transcobalamin–cobalamin complex [4]. This confirmed the essential role of *CD320* in cellular cobalamin transport. Clinical phenotypes are highly variable, ranging from biochemical abnormalities detected during NBS to developmental and neuropsychological phenotypes, with many affected individuals remaining largely asymptomatic [4]. Knockout of *CD320* in mice confirmed its importance in cobalamin and tissue vitamin B_12_ homeostasis [5,6].

Here, we report a novel recessive missense candidate variant and adjacent synonymous candidate variant in *CD320* segregating in a consanguineous family that is associated with cobalamin transport deficiency, along with an expanded phenotype to potentially include microcephaly.

## 2. Methods

### 2.1. Patient Recruitment and Ethical Approval

This study was approved by the Institutional Review Board of the College of Applied Medical Sciences at Taibah University (approval no. 2023/146/103 MLT), and conducted in accordance with the Declaration of Helsinki. A family with a patient affected by a rare genetic disease was recruited for this study. The parents provided written informed consent for themselves and on behalf of their children (the proband and unaffected siblings) prior to participation in this study. Clinical examination of the proband was conducted by the responsible physicians at Alhada Armed Forces Hospital in Taif, Saudi Arabia. 

### 2.2. DNA Extraction

Peripheral blood was collected from family members in ethylenediaminetetraacetic acid tubes and genomic DNA was extracted using a QiaAmp DNA Mini Kit (cat. no. 51306; Qiagen, Hilden, Germany) according to the manufacturer’s instructions.

### 2.3. Metabolic Analysis

The proband underwent routine NBS of a dried blood spot using tandem mass spectrometry according to the manufacturer’s protocol. Urine organic acid analysis was carried out using gas chromatography–mass spectrometry. Confirmatory biochemical analysis was performed on plasma using liquid chromatography/tandem mass spectrometry.

### 2.4. Whole Exome Sequencing

**Figure 1:**
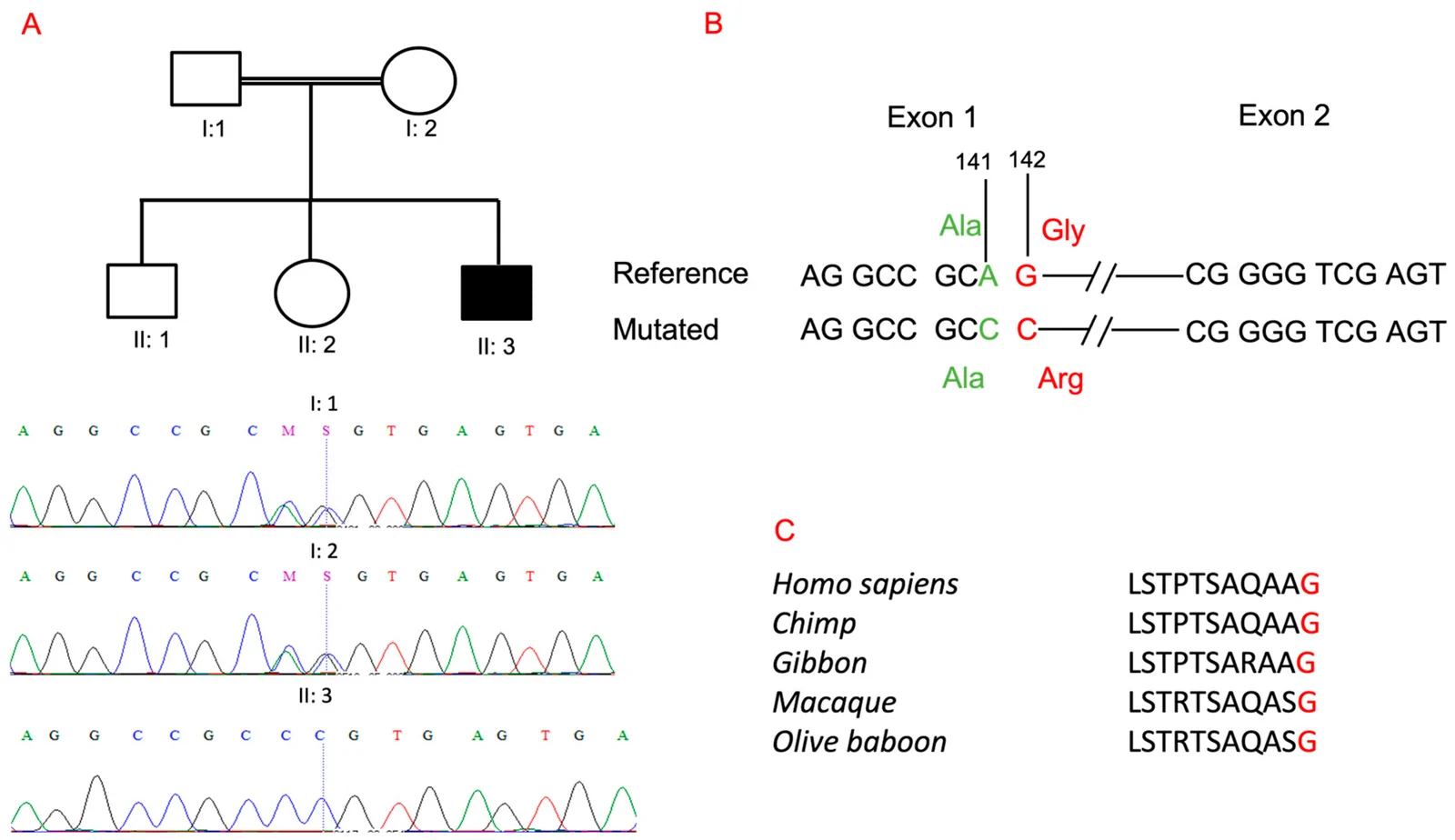
The family pedigree, Sanger results, Exon junction cartoon and amino acid orthologue of a homozygous *CD320* variant. (**A**) The pedigree and Sanger sequence result for the proband, siblings and their consanguineous parents. The partial chromatogram result highlights the faint line and shows the homozygous variant in the proband (II.3) and the heterozygous parents (I:1 and I:2). (**B**) Simplified figure to show the junction missense variant in red (c.142G>C, p.Gly48Arg) and the adjacent synonyms variant in green (c.141A>C, p.Ala47=). (**C**) Comparison of amino acid sequences between the species, where the red-highlighted amino acids show amino acids of interest.

Whole-exome sequencing (WES) was carried out for the genomic DNA from the proband (Affected II.3, Figure 1A). The sequencing library was prepared using a SureSelect Human All Exon Kit V6 (Agilent, Santa Clara, CA, USA). Sequencing was generated on the HiSeq4000 platform (Illumina, San Diego, CA, USA) with an average read depth of approximately 100×. Next, raw paired-end FASTQ sequence reads were adapted, trimmed and subjected to quality control using commercial bioinformatics pipelines. Then, high-quality reads were mapped to the human reference genome assembly (GRCh37) using BWA-MEM (v 0.7.15) and variants were called using GATK4 (version 4.2). Finally, the identified variants were annotated and filtered using the commercial Clinical Insight tools (QIAGEN, Hilden, Germany). The percentage of targeted bases covered at depth of more than or equal to 20× was 98.7%.

Based on the family pedigree (Figure 1A), an autosomal recessive pattern of inheritance was assumed and homozygous variants were prioritized during the filtering process. Variants were called with a minimum read depth of 20. Variants with a minimum allele frequency of less than 0.01% were considered for further analysis. Copy number variants (CNV) were not performed. Noncoding and splice variant were assessed based on splice-region variants captured using an enrichment kit. Analysis runs of homozygosity regions were carried out for selected regions (6q24, 7, 11p15.5, 14q32, 15q11q13, 20q13 and 20), but other regions were not tested. Variant nomenclature followed the recommendations of the Human Genome Variation Society (HGVS), and variant classification was performed in accordance with the guidelines of the American College of Medical Genetics and Genomics (ACMG) [7].

### 2.5. Sanger Sequencing and Variant Prediction Tools

The candidate variant identified by WES was validated by Sanger sequencing in the proband and their family members. Primers were designed using Primer 3 (http://primer3.ut.ee/, accessed on 10 May 2026). The reference sequence for the *CD320* gene was downloaded from the Ensemble Genome Browser (https://m.ensembl.org/, accessed on 10 May 2026). Sequence alignment and analysis were carried out using Clustal Omega [8]. The potential impact of the variant was assessed using the combined annotation-dependent depletion (CADD) score (GRCh38-v1.7) [9]. Further in silico prediction tool dbscSNV was used to predict the pathogenicity effect on the protein. Variant frequency and novelty were assessed using several databases, including the Genome Aggregation Database (gnomAD), Exome Sequencing Project (ESP), and the 1000 Genomes Project [10,11,12]. Clinical significance was further evaluated using ClinVar (https://www.ncbi.nlm.nih.gov/clinvar, accessed on 26 May 2026) and the Human Genome Mutation Database (HGMD) [11].

## 3. Results

### 3.1. Clinical Presentation

A male patient was born to a consanguineous family with a 43-year-old mother at 41 weeks of gestation with spontaneous natural delivery. At birth, the patient showed low birth weight (2.39 kg), borderline length (48 cm), and severe microcephaly (head circumference of 28 cm, reference: 33–35 cm). NBS showed elevated C3 level (13 µmol/L; reference: ≤8 µmol/L), elevated C3/C2 ratio (0.8; reference: ≤0.2) and elevated C3/C16 ratio (5.7; reference: ≤2). Urine organic acid analysis suggested a high level of MMA. Plasma metabolic investigation showed an elevated MMA level (36,300 nmol/L; reference: 55–347 nmol/L), while the plasma homocysteine level was within the normal range at 9.6 µmol/L. Blood gas analysis showed mild metabolic acidosis (pH 7.32; reference: 7.35–7.45), low partial pressure of carbon dioxide (27 mmgh; reference: 35–45 mmgh), low bicarbonate (13.9 mmol/L; reference: 22–26 mmol/L), extremely low base excess (−12 mmol/L; reference: −2 to +2 mmol/L) and elevated serum lactate (6.8 mmol/L; reference: 0.2–2.2 mmol/L). Blood investigation showed pancytopenia. Serum cobalamin level was measured several times and was within the normal range. Additionally, the mother’s serum cobalamin level was normal. Neuroimaging workup during the neonatal period, including cranial ultrasound and non-contrast computed tomography of the brain showed no remarkable abnormalities.

The patient was managed with specialized metabolic formula and levocarnitine supplementation for one year before the genetic investigation to avoid acute metabolic decompensation. During regular follow-up, the patient did not show recurrent metabolic crises or other significant metabolic decompensation. Head circumference was measured at visits and showed gradual interval growth from 33 cm to 35 cm, 36 cm, and 38 cm.

After one year, the patient was admitted for further metabolic evaluation and monitoring. During admission, the patient was transitioned to a regular diet while both levocarnitine supplementation and specialized metabolic formula were temporarily withheld. The patient tolerated the transition well, with no signs or symptoms of metabolic decompensation. Serial blood gas analyses were performed every 6 h and remained within normal limits throughout hospitalization. Additionally, the patient received one intramuscular injection of hydroxocobalamin dose (1 mg/mL). Physical examination showed persistent microcephaly with a sloping forehead, with a head circumference of 40 cm, which remained below the third percentile for his age. The patient showed an increase in length (66 cm) and weight (9.5 kg) during follow-up with both measurements remaining at the 15th percentile. The repeated serum MMA level was decreased to 672 nmol/L (reference: 55–347 nmol/L) and the plasma homocysteine level remained normal. Additionally, pancytopenia was resolved. No apparent neurological abnormalities were identified, and hearing and vision were unremarkable.

### 3.2. Genetic Analysis

WES identified a homozygous missense candidate variant at the *CD320* gene (NM_016579.3: c.142G>C, p.Gly48Arg) with a CADD score of 33. This variant is located at the amino acid glycine at position 48 and is replaced by arginine. This variant is located at the first nucleotide of the last amino acid of exon 1. It predicts a change at the donor site 1 bp downstream of the splice site. The splice prediction tool dbscSNV indicates a pathogenic variant, with an ADA score of 0.9999.

The identified variant was not found in the gnomAD database and is absent in the Exome Aggregation Consortium data and the 1000 Genome Project data [10,11,12], and a local database out of 200 individuals. It is also located in a region highly conserved regions across species (Figure 1C). The variant was confirmed using Sanger sequencing (Figure 1A). Additionally, an adjacent synonymous homozygous candidate variant was identified using Sanger sequencing that could affect splicing because it is located at the penultimate nucleotide (NM_016579.3: c.141A>C, p.Ala47=; dbSNP: rs1599625837). The allele frequency for this synonymous variant was rare, with less than 0.01 (0.000001961). However, the prediction tools find this variant benign or neutral (Table S1). Sanger sequencing of both variants in the parents and the unaffected siblings confirmed they were heterozygous.

## 4. Discussion

Pathogenic variants in the *CD320* gene cause impaired cellular cobalamin uptake despite systemic vitamin B_12_ availability [3,4]. In the current study, a novel recessive missense candidate variant in the *CD320* gene, located at the last amino acid of exon one, is reported. Another adjacent synonymous candidate variant is reported. Similar to the reported cases, our patient showed elevated C3 and MMA, supporting disruption of intracellular cobalamin metabolism. He also experienced an episode of metabolic acidosis. Notably, our patient also presented with microcephaly, a feature that has not been reported in others with *CD320* variants. These findings provide evidence supporting the likely pathogenic role of the identified variant and further emphasize the importance of *CD320* in intracellular cobalamin transport.

Both variants are located near the exon/intron boundary. The missense variant (Gly48Arg) was predicted to be pathogenic. However, several in silico prediction tools consistently predicted the adjacent synonymous variant (Aln47=) to be benign or neutral, with no predicted impact on splicing. The c.142G>C (p.Gly48Arg) variant is located in the last nucleotide of exon 1. This nucleotide represents the first base of the Gly 48 codon, while the remaining two nucleotides of the codon are located at the beginning of exon 2. Thus, the Gly48 codon spans the exon1–exon 2 boundary. The synonymous variant, c141A>C, occurs upstream at the third nucleotide of the preceding codon (Ala47), which is also located at exon 1 (Figure 1B). Both variants could not be assessed as a combined haplotype using the prediction tools in the current study. Therefore, the pathogenic effect cannot be independently assigned to the c.142G>C variant with confidence. In fact, additional functional studies at the protein level are necessary to identify the individual or combined effect of the two variants.

The c.142G>C (p.Gly48Arg) variant was submitted to ClinVar by King Faisal Specialist Hospital and Research Centre in Riyadh, Saudi Arabia, which stated that several individuals were identified as heterozygous for it (VCV003393196.1). Another indel variant (NM_016579.4: c.141_142delinsCC, p.Gly48Arg) at the same amino acid was submitted to ClinVar by Labcorp Genetics (formerly Invitae, Labcorp (VCV004714726)), which is not reported in population database. However, information on phenotypes and biochemical findings remains insufficient. Therefore, both variants were classified as variants of uncertain significance (VUS).

The identified missense variant (NM_016579.3: c.142G>C, p.Gly48Arg) was assessed in accordance with the ACMG guidelines for variant classification [7]. It is not detected in large population databases, meeting the moderate evidence criterion (PM2). In silico prediction tools predicted a pathogenic effect on protein level, meeting the support evidence criterion (PP3). Therefore, based on one moderate evidence criterion (PM2) and three supporting criteria (PP3), the variant is classified as VUS. Thus, to upgrade the variant’s classification, additional affected individuals and functional studies would be required.

Extensive functional studies in several patient fibroblasts with a deletion variant in *CD320* (p.E88del) established *CD320* as a critical component of cellular cobalamin transport. It showed a reduced ability to bind and uptake the transcobalamin-bound complex by p.E88del isoform [3,4]. Further studies showed impaired cellular uptake of transcobalamin-bound cobalamin, which reduced intracellular vitamin B_12_ availability. In addition, methylmalonyl-CoA mutase and methionine synthase, two main cobalamin-dependent enzymes that require vitamin B_12_ as a cofactor, showed reduced activities [4]. This is because when hydroxocobalamin supplementation was added to the cell culture, vitamin B_12_ uptake was increased [4].

Several studies have reported variable phenotypes among individuals carrying the deletion variant (p.E88del) in the *CD320*. All were identified through NBS with elevated C3 and plasma MMA and were phenotypically asymptomatic [3,13], as confirmed by a long-term follow-up of two individuals from different families [13]. Both studies used different forms of cobalamin treatment at different doses and times [3,13]. Another case with the same variant showed a patient with elevated MMA and hyperhomocysteinemia, and had bilateral central retinal artery occlusions [14]. However, a recent study identified several cases with up to 15 years of follow-up [4]. It showed variable phenotypes ranging from transient elevation of C3 and MMA with no significant phenotype to several neurological findings such as deficient language acquisition and working memory, attention deficit hyperactivity disorder, autism spectrum disorder, and anxiety [4]. These suggest alternative cobalamin uptake pathways may compensate for impaired *CD320* function in the absence of transcobalamin defects.

*CD320* has been knocked out in several animal models to identify its function and provide insights into the phenotypic variability observed in humans. *CD320* knockout mice were viable and showed variable phenotypes [5,6,15]. All mice showed elevated methylmalonic acid and homocysteine levels. In fact, when a knocked-out mouse was kept under normal dietary conditions, it showed a healthy life. In restricted vitamin B_12_ conditions, however, mice developed severe macrocytic anemia and reproductive defects [15]. This phenotype was observed in our patient, who showed pancytopenia. Despite anemia phenotypes, the knockout mice did not exhibit obvious neurological phenotypes [15]. However, another study found that mice lacking *CD320* generated by Shao-Chiang Lai (2013) had significantly reduced cobalamin levels in the brain, accompanied by elevated brain methylmalonic acid and homocysteine concentrations, and reduced S-adenosylmethionine availability [6]. In another study, knockout mice had 90% lower cobalamin level and 40% lower global DNA methylation in the brain than control mice [5]. These findings suggest that the brain may be particularly vulnerable to impaired *CD320*-mediated cobalamin uptake. Given the essential role of vitamin B_12_ in DNA synthesis, cellular proliferation, and epigenetic regulation, these observations provide a biologically plausible mechanism linking *CD320* dysfunction to the microcephaly observed in our patient; however, a direct causal relationship cannot be established from a single case.

Before obtaining the genetic results, the patient presented with clinical and biochemical features that strongly mimicked classical isolated methylmalonic acidemia. Consequently, to prevent acute metabolic decompensation, he was immediately started on empirical management following standard methylmalonic acidemia guidelines, which included a specialized protein-restricted metabolic formula to limit precursor amino acids and levocarnitine to facilitate acylcarnitine excretion. After WES identified the recessive missense variant in *CD320*, the treatment strategy was shifted toward targeted single-dose hydroxocobalamin therapy [16]. However, safely transitioning the patient from a specialized metabolic diet to a normal regular diet required elective hospitalization for close clinical observation and serial biochemical monitoring to ensure metabolic stability upon protein reintroduction, which accounts for the timeline observed. The family received genetic counseling about the diagnosis and its autosomal recessive inheritance. They were also informed about the recurrence risk in future pregnancies and the risk of chromosomal abnormalities regarding the mother’s age. They were also recommended to follow up with the in vitro fertilization clinic for future pregnancies.

As a limitation of this study, the CNV calling was not carried out in the WES analysis. We cannot completely exclude CNV, structural abnormalities, other pathogenic or likely pathogenic variants located in the genomic region that were not captured. Therefore, the possible association between microcephaly and the CD320-cobalamin deficiency might be interpreted carefully, and a definitive causal relationship cannot be established based on the current findings.

In conclusion, this study expands the molecular spectrum of *CD320*-related disorders by identifying a novel recessive missense candidate variant and adjacent synonymous candidate variant. Furthermore, the presence of microcephaly suggests a potential association with impaired CD320-mediated cobalamin uptake and may broaden its associated phenotypic features.

## Data Availability

Data sharing is available upon request to the author.

## Supplementary Materials

The following supporting information can be downloaded at https://www.mdpi.com/article/10.3390/genes17091108/s1: Table S1: Individual in silico prediction for both variants [17,18,19,20,21].

## Abbreviations

ACMG	American College of Medical Genetics and Genomics
CADD	Combined Annotation-Dependent Depletion
HGVS	Human Genome Variation Society
MMA	Methylmalonic Acid
NBS	Newborn Screening
C3	Propionylcarnitine
CD320	Transcobalamin Receptor
WES	Whole-Exome Sequencing
