# Supplementary material for: Identification of CD320-Associated Cobalamin Transport Deficiency Through Newborn Screening Caused by Novel Homozygous Candidate Variants
Source: Genes (Basel). 2026 Sep 12;17(9):1108. doi: 10.3390/genes17091108 (PMC13606811; doi:10.3390/genes17091108)
Supplement: Supplementary file 1 [file genes-17-01108-s001.zip › genes-4523251-supplementary.pdf]

Supplementary Table S1

Name	NM_016579.3: c.141A>C, p.Ala47=		NM_016579.3: c.142G>C, p.Gly48Arg		Reference Pathogenic Cutoff (Threshold)
	**Prediction**	**Numerical scores**	**Prediction**	**Numerical scores**	
CADD (v1.6) [17]	Benign	12	pathogenic	33	≥ 20 (Top 1% most deleterious)
dbscSNV1_ADA [18]	Benign	0.12	pathogenic	1	≥ 0.708
dbscSNV1_RF [18]	Benign	0.58	pathogenic	0.97	≥ 0.515
SpliceAI score (max) v 0.21 [19]	Benign	0.02	Benign	0.12	≥ 0.20 (High Sensitivity)≥ 0.50 (Recommended/Standard)≥ 0.80 (High Specificity)
SIFT (v6.2.0) [20]	NA	NA	Deleterious	0	≤ 0.05
PolyPhen2 (v): HDivPred [21]	NA	NA	Probably damaging	1	≥ 0.957 (Probably Damaging)
PolyPhen2 (v): HVarPred [21]	NA	NA	Probably damaging	0.974	≥ 0.909 (Probably Damaging)
BayesDel_noAF [21]	Benign	-0.7	NA	NA	≥ -0.0570105

Table S1. Individual *in silico* prediction for both variants.

1. Rentzsch P, Schubach M, Shendure J, Kircher M. CADD-Splice-improving genome-wide variant effect prediction using deep learning-derived splice scores. Genome Med. 2021;13(1):31.
2. Jian X, Boerwinkle E, Liu X. In silico prediction of splice-altering single nucleotide variants in the human genome. Nucleic Acids Res. 2014;42(22):13534-44.
3. Jaganathan K, Kyriazopoulou Panagiotopoulou S, McRae JF, Darbandi SF, Knowles D, Li YI, et al. Predicting Splicing from Primary Sequence with Deep Learning. Cell. 2019;176(3):535-48.e24.
4. Sim NL, Kumar P, Hu J, Henikoff S, Schneider G, Ng PC. SIFT web server: predicting effects of amino acid substitutions on proteins. Nucleic Acids Res. 2012;40(Web Server issue):W452-7.
5. Adzhubei IA, Schmidt S, Peshkin L, Ramensky VE, Gerasimova A, Bork P, et al. A method and server for predicting damaging missense mutations. Nat Methods. 2010;7(4):248-9.
